# Asbestos bodies count and morphometry in bulk lung tissue samples by non-invasive X-ray micro-tomography

**DOI:** 10.1038/s41598-021-90057-1

**Published:** 2021-05-19

**Authors:** Fabrizio Bardelli, Francesco Brun, Silvana Capella, Donata Bellis, Claudia Cippitelli, Alessia Cedola, Elena Belluso

**Affiliations:** 1 Institute of Nanotechnology, CNR-Nanotec, Roma, Italy; 2 Department of Engineering and Architecture, University of Trieste, Trieste, Italy; 3 Department of Earth Sciences, University of Torino, Torino, Italy; 4 Interdepartmental Centre for the Study of Asbestos and Other Toxic Particulate, University of Torino, Torino, Italy; 5 Department of Pathological Anatomy, Ospedale degli Infermi, Biella, Italy; 6 Division of Respiratory Diseases, Department of Clinical and Molecular Medicine, Sant’Andrea Hospital, Sapienza University, Roma, Italy

**Keywords:** Environmental sciences, Natural hazards, Diseases, Health care

## Abstract

The number of the Asbestos Bodies (AB), i.e. asbestos that developed an iron-protein coating during its permanence in biological tissues, is one of the most accessible markers of asbestos exposure in individuals. The approaches developed to perform AB count in biological tissues are based on the manual examination of tissue digests or histological sections by means of light or electron microscopies. Although these approaches are well established and relatively accessible, manual examination is time-consuming and can be reader-dependent. Besides, approximations are applied because of the limitations of 2D readings and to speed up manual counts. In addition, sample preparation using tissue digests require an amount of tissue that can only be obtained by invasive surgery or *post-mortem* sampling. In this paper, we propose a new approach to AB counting based on non-destructive 3D imaging, which has the potential to overcome most of the limitations of conventional approaches. This method allows automating the AB count and determining their morphometry distribution in bulk tissue samples (ideally non-invasive needle biopsies), with minimal sample preparation and avoiding approximations. Although the results are promising, additional testing on a larger number of AB-containing biological samples would be required to fully validate the method.

## Introduction

Asbestos is well known to induce several lung diseases, including asbestosis, pleural plaques, malignant mesothelioma, and lung cancer^1–4^. Although the production and commercialization of asbestos is strictly regulated world-wide, and it is officially banned in most countries, the weathering of existing asbestos products and naturally occurring asbestos make asbestos a current global health risk, with hundreds of thousands deaths per year^5,6^. In addition, the peak of mortality is yet to come because of the long latency time of mesothelioma^7–11^, which ranges between 20 and 40 years, on average^12^.

Asbestos bodies (AB) are the result of a biomineralization process taking place on asbestos in biological tissues. This process causes Fe-proteins and other organic and inorganic material to cluster around asbestos. Asbestos bodies were believed to be the organism effort to isolate the foreign fibres from the surrounding tissues^13,14^ to prevent the development of asbestos-related diseases. However, later researchers suggested that they might enhance the cytotoxic properties of asbestos. In particular, based on the results of in vitro (cell cultures) and in vivo (animal) studies, it was showed that AB are able to induce the generation of free radicals^15,16^, double strand breaks in the DNA^17^, and that the iron contained in the coating is catalytically active^15,18,19^.

Beyond their possible role in the pathogenesis of asbestos-related diseases, the number of AB in the lungs is the most accessible indicator to assess the asbestos exposure in individuals^20^. However, the AB number in the lungs is a coarse estimate of the actual asbestos fibre burden in the lungs^21^. In fact, the number of AB can increase after the last exposure to asbestos, as, in time, more fibres become coated. Moreover, it was observed that the AB preferentially form on the longer (> 5–10 um), thinner (< 0.5 um)^22^, and more biopersistent amphiboles fibres (amosite, antophyllite, crocidolite, tremolite, and actinolite), which were also observed to be the most carcinogenic^22^. In addition, though the AB do form on chrysotile fibres too^23^, these can undergo to fragmentation, so that the organism’s clearance mechanisms are able to remove most of them. As a result, chrysotile usually accounts for a minor fraction of asbestos in the lungs^24,25^. Bodies similar to the AB can also form on non-asbestos fibres (e.g. mica and rutile fibres), and, in this case, the term ferruginous body is used. However, even when contamination by other fibres is present along with asbestos exposure, the AB usually account for more than 90% of all coated fibres^20,26^.

In the years, several approaches have been established and refined to extract and estimate the number of AB in unit of weight (g) or volume (cm^3^) of wet or dry autoptic (post-mortem) lung tissue samples, broncho-alveolar lavage (BAL) and sputum samples, needle, transbronchial or thoracoscopic biopsy samples^27–31^.

Quantitative analysis performed on lung tissues is the standard method to assess the AB concentration in the lungs. Nevertheless, it can only be performed after autopsy, or it requires a lung biopsy. Needle biopsies, however, produce an amount of biological tissue that is usually insufficient for AB count with conventional methods, and thoracoscopic biopsies require invasive surgery. On the other hand, BAL and sputum samples only reveal AB from the alveolar airspace, and AB are not present in sputum when their burden is lower than ~ 10,000 AB/g of dry lung tissue^31^.

Besides, sample preparation is one of the critical point of conventional AB counting approaches. Depending on the sampling method, lung tissues can prepared in two ways. In the first, the tissue is removed by chemical digestion or ashing, followed by a variable number of ultrasonication, filtration, washing (with distilled water and hydrochloric acid), and centrifugation steps to recover and enrich the AB in the resulting precipitate. This is deposited on sample holders (grids) for transmission electron microscopy (TEM), or on porous membranes for observation with optical microscopy (OM) or scanning electron microscopy (SEM). The second approach consists in fixing the tissue samples (usually in formalin or alcohol), and then embedding them in paraffin. A microtome is then used to cut thin slices (usually 3–6 µm-thick) suitable for OM observations from the paraffin-embedded tissue blocks. Thinner slices (50–100 nm-thick), suitable for TEM observations, are obtained by using an ultra-microtome and epoxy-embedded tissue blocks.

Each of the above sample preparation approaches has limitations and disadvantages. For example digestion in potassium hydroxide, sodium hypochlorite or formamide, or low temperature ashing have been shown to alter or destroy the AB^32,33^. The use of ultrasound to help removing the tissue may fragment the AB, altering their size distribution and possibly resulting in higher counts^21^. Some AB may be lost during centrifugation and filtration steps that are used to concentrate their content^21^.

Another critical aspect of conventional methods is that they eventually all rely on the observations of a human reader for the actual AB count by optical or electron microscopies (OM, SEM, or TEM). Because manual observations can be extremely time-consuming, approximations are often applied. These usually consist of counting the AB on areas of the AB supports (porous membrane, OM slide, or TEM grid) much smaller than their whole area, and extend the result on the rest of the area assuming uniform distribution. Optical microscopy is undoubtedly the most accessible and less time consuming among the reading techniques. In addition, sample preparation for OM observations is easier and larger areas of tissue slices (~ 1–4 cm^2^) can be probed because of the lower magnification achievable. Electron microscopies allow detecting also uncoated fibres and determining their chemical composition by energy dispersive spectroscopy (EDS)^34^. Transmission electron microscopy can also reveal their crystallographic structure and level of crystallinity by performing selected area electron diffraction (SAED)^34,35^. On the other hand, electron microscopies are less accessible and require complex and invasive sample preparation, as they always require the removal of the biological tissue to achieve good quality images (contrary to OM, which can be performed also on histological sections). Besides, the observations are more time consuming because the probed area is much smaller (~ 100–1000 µm^2^, depending on the magnification for SEM, less for TEM). A further downside is that the results obtained have higher variability between laboratories and are therefore more difficult to compare.

In this paper, we report on a novel approach to AB counting based on X-ray phase-contrast micro-tomography (XPCµT) and requiring non-invasive sample preparation, which may overcome most of the limitations of the conventional approaches discussed above. X-ray tomography is a non-destructive, tridimensional imaging technique that, thanks to the large penetration depth of hard X-rays (> 1 keV), can be applied to macroscopic (bulk) biological samples up to few centimeters in size. Although radiation damage to the samples may occur with the high flux provided by 3rd generation synchrotron source (by heating), negligible damage is expected when using monochromatic and relatively high-energy X-ray beams (> 10 keV) on thin (< 10 mm) biological samples, as is the case of the measurements reported in this work. Furthermore, in contrast with conventional methods, X-ray imaging requires minimal sample preparation (virtually none). The combination of 3D imaging and automated counting routines allows to count all AB in a given lung tissue volume, without any a priori knowledge of the AB morphometry, which is instead required when estimating the number of AB in 2D images (see “Materials and methods” section). A crucial advantage of performing AB count with automated routines is that the counting is reader-independent and much less time-consuming, particularly in case of samples with high content of asbestos. For best results, in this proof-of-concept work synchrotron radiation was used as hard X-ray source to perform micro-tomography. However, efficient commercial laboratory X-ray sources and tomographs with the ability to work in the hard X-rays energy range and reach sub-micrometric resolutions are becoming available. In addition, in this work, phase-contrast tomography was used in place of conventional absorption tomography, with the aim of imaging the lung tissue along with the AB. However, as AB have a relatively high mass density with respect to the lung tissue, conventional absorption tomography would suffice to perform AB counts.

## Results

**Figure 1:**
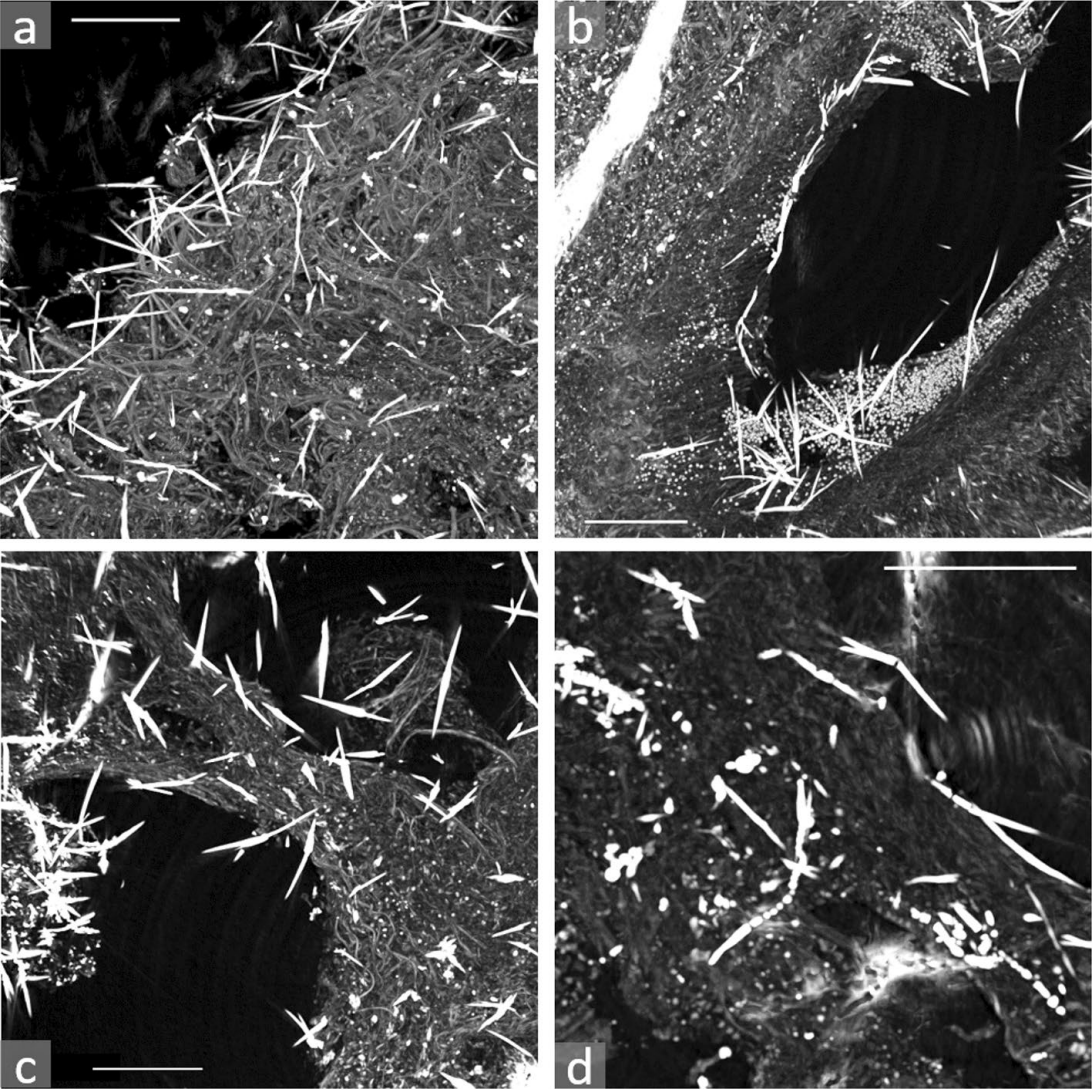
XPCµT data acquired at the I13-2 beamline at the Diamond light XPCµT data acquired at the I13-2 beamline at the Diamond light source on lung tissue fragments embedded in paraffin. Brighter grey levels correspond to more absorbing (i.e. denser) material. For ease of view, the volumes are shown as projections on a plane of 300 slices, each 0.332 μm-thick (total thickness ~ 99 μm). In all images, several AB can be clearly identified as the white elongated object. (**a**) *Sample A* AB in are visible along with lung structures, such as bronchioles and alveoli. (**b**) *Sample C* an alveolar sac with AB accumulating on its walls, preferentially at its end, where hypercellularity occurs. (**c**) *Sample D* alveoli or bronchioles with AB penetrating the lung parenchyma. (**d**) *Sample A* magnification showing a variety of typical AB with sheatlike or beaded coatings. In all images, the scale bars are 100 μm long and the pixel size is 0.332 × 0.332 μm^2^.

X-ray phase-contrast micro-tomography was performed on fragments of formalin-fixed paraffin-embedded lung tissue blocks that belonged to four workers subjected to prolonged occupational exposure to asbestos (Table 1). Three tissue fragments (indicated as volumes, in the following) of size 0.83 × 0.83 × 0.7 mm^3^ were imaged for each sample. The size of the voxels (i.e. 3D pixels) was ~ 0.332 × 0.332 × 0.332 μm^3^. The spatial resolution was roughly double that of the voxel size^37^. Representative tomographic data of lung samples are shown in Fig. 1, where volumes of ~ 0.495 × 0.495 × 0.1 mm^3^ are shown projected on a plane for easy of view. The images are in grey levels: darkest levels correspond to lowest densities, brightest to highest ones. The AB can be clearly identified as the white (i.e. higher density), high aspect ratio objects. The dark round-shaped holes have dimensions compatible with lung structures, such as respiratory bronchioles, alveolar ducts, atriums, or alveolar sacs (average diameter of 250–300 μm, when inflated). Thanks to the phase-contrast mode that enhances the small density variations of the biological tissues, the capillary beds surrounding the alveolar sacs can also be seen (Fig. 1a). The ubiquity of the AB in the imaged volumes reflects the high asbestos burden in the tissue samples, but is also due to the relatively large equivalent thickness of the volumes shown in Fig. 1: about 100 μm-thick compared to the 3–6 μm-thick histological sections commonly used in optical microscopy analyses. The tomograms reveal that the AB accumulate on the walls of the alveoli, where hypercellularity occurs, as can be seen at the end of the alveolar sac shown in Fig. 1b. These cells are likely to be mononuclear macrophages or neutrophils that migrated in AB-rich areas. Higher magnification (Fig. 1d) reveals the typical morphology of the AB, i.e. the sheath-like protein layer that develops around the asbestos fibres, which can be often beaded or segmented (Fig. 3d)^32,38,39^.

**Table 1:** Samples’ description.

Case	Age	Sex	Occupation	Exposure period (years)	Disease
A	80	F	Fibre cement plant	> 10	AS (grade 4), PP, LC
B	81	M	Fibre cement plant	27 (1955–1982)	AS (grade 3), PP, MM
C	85	M	Steel plant	22 (1962–1984)	AS (grade 4), PP, MM
D	87	M	Asbestos mine	35	AS (grade 4), PP, SS, SC

*AS* asbestosis (grading established according to Craighead et al*.* 1982^36^, *PP* pleural plaques, *MM* malignant mesothelioma, *LC* lung cancer, *SS* siderosis, *SC* silicatosis.

### Asbestos bodies’ count

The amount of AB per cubic centimeter of lung tissue (AB/cm^3^) as determined by X-ray tomography is reported in Table 2 for each sample. The numbers reported in Table 2 represent the average of three volumes for each sample. The standard deviations associated to the AB numbers are as high as 50% of the values, reflecting the heterogeneity of the AB distribution in the lung tissues. The number of AB/cm^3^ falls within the same order of magnitude for all samples (2.60–1.43 × 10^6^/cm^3^).

**Table 2:** Average number of AB and morphometry (length, width, and volume) for each sample.

Sample	AB (cm^−3^)	AB* (g_dw_^−1^)	AB(OM) (g_dw_^−1^)	AB(SEM) (g_dw_^−1^)	Length (μm)	Width (μm)	Volume (μm^3^)
A	2.60 ± 0.64 × 10^6^	12.4 × 10^6^	48.0 × 10^3^	11.9 × 10^6^	22.5	3.6	153
B	2.35 ± 0.50 × 10^6^	11.1 × 10^6^	23.8 × 10^3^	0.36 × 10^6^	23.6	3.3	133
C	1.43 ± 0.98 × 10^6^	6.80 × 10^6^	34.4 × 10^3^	18.4 × 10^3^	20.8	3.5	131
D	2.07 ± 1.17 × 10^6^	9.84 × 10^6^	56.1 × 10^3^	12.7 × 10^3^	19.4	3.6	107

The number of AB derived from XPCµT measurements is reported per cubic centimeter (AB/cm^3^) and converted per gram of dry weight of tissue (AB*/g_dw_), using Eq. (1). The number of AB estimated by OM on digested tissues samples (AB/g_dw_) are also reported for comparison. The errors on the numbers of AB correspond to the standard deviation calculated on three different volumes for each sample.

The relation between AB/cm^3^ and AB/g_dw_ was determined by Roggli and Pratt^40^ and is reported in the following equation:

1 $${N}_{gdw}={N}_{g}\times {D}_{dw}=\frac{{N}_{c}}{{V}_{c}\times {V}_{s}\times {O}_{c}\times {R}_{wv}},$$

where *N*_*gdw*_ is the number of AB per gram of dry weight of lung tissue (i.e. the standard way to represent the AB number in the tissues), *N*_*g*_ is the number of AB per gram of wet weight of lung tissue, *D*_*dw*_ is the wet to dry weight conversion factor, *N*_*c*_ is the number of AB in a given formalin-fixed paraffin-embedded lung tissue volume (in the original paper by Roggli and Pratt^40^, *N*_*c*_ was the number of AB in an iron-stained histological section), *V*_*c*_ is the lung tissue volume (in the original paper it represented the area times the thickness of the lung tissue section), *O*_*c*_ is the orientation correction factor, *R*_*wv*_ is the wet weight to volume ratio of the fixed lung tissue, and *V*_*s*_ is the shrinkage correction factor from formalin fixed to paraffin-embedded tissue. The last factors (*R*_*wv*_ and *V*_*s*_) were assumed to be the same as those calculated by Roggli and Pratt^40^ (respectively 0.916 g/cm^3^ and 2.3, dimensionless). The wet to dry tissue correction factors (*D*_*dw*_) were calculated and resulted close to 10. The sample volume (*V*_*c*_) can be calculated from tomographic data with sub-micrometric precision (corresponding to the size of the voxels).

The orientation correction factor (*O*_*c*_) was introduced by Roggli and Pratt^40^ in Eq. (1) to take into account AB with out-of-plane orientations in tissue thin-sections suitable for OM observations (3–6 µm-thick). This correction is necessary to prevent counting the same AB in subsequent tissue sections when the length of its projection orthogonal to the section surface exceeds the section thickness. This factor depends on the length of the AB and on the angle between the AB and the section surface: assuming a uniform distribution of the AB orientation respect to the section surface, *O*_*c*_ can be calculated if the average AB length is known, leading to an approximated AB count. In this framework, the advantage of a tomographic approach is evident, as for 3D data *O*_*c*_ in Eq. (1) is equal to unity, independently of the AB length and orientation, and the AB count is now exact.

The numbers of AB per gram of dry tissue (AB*/g_dw_, Table 2) obtained converting those per tissue volume (AB/cm^3^, Table 2) using Eq. (1), fall within the same order of magnitude for all samples. The same occurs to the numbers of AB calculated by OM on tissue digests (AB/g_dw_), although these are three orders of magnitude lower (Table 2). Unfortunately, due to the low amount of lung tissue available it was not possible to perform duplicate or triplicate measurements for the digested samples. Therefore, standard deviations are not available for conventional OM or SEM count and no clear relation between the trends of the two series of values can be determined. Scanning electron microscopy readings of digested samples, on the other hand, returned numbers differing by more than three order of magnitudes between samples A and B, and samples C and D (the SEM values reported for sample A and B are closer to those calculated by micro-tomography, Table 2).

### Asbestos bodies’ morphometry

**Figure 2:**
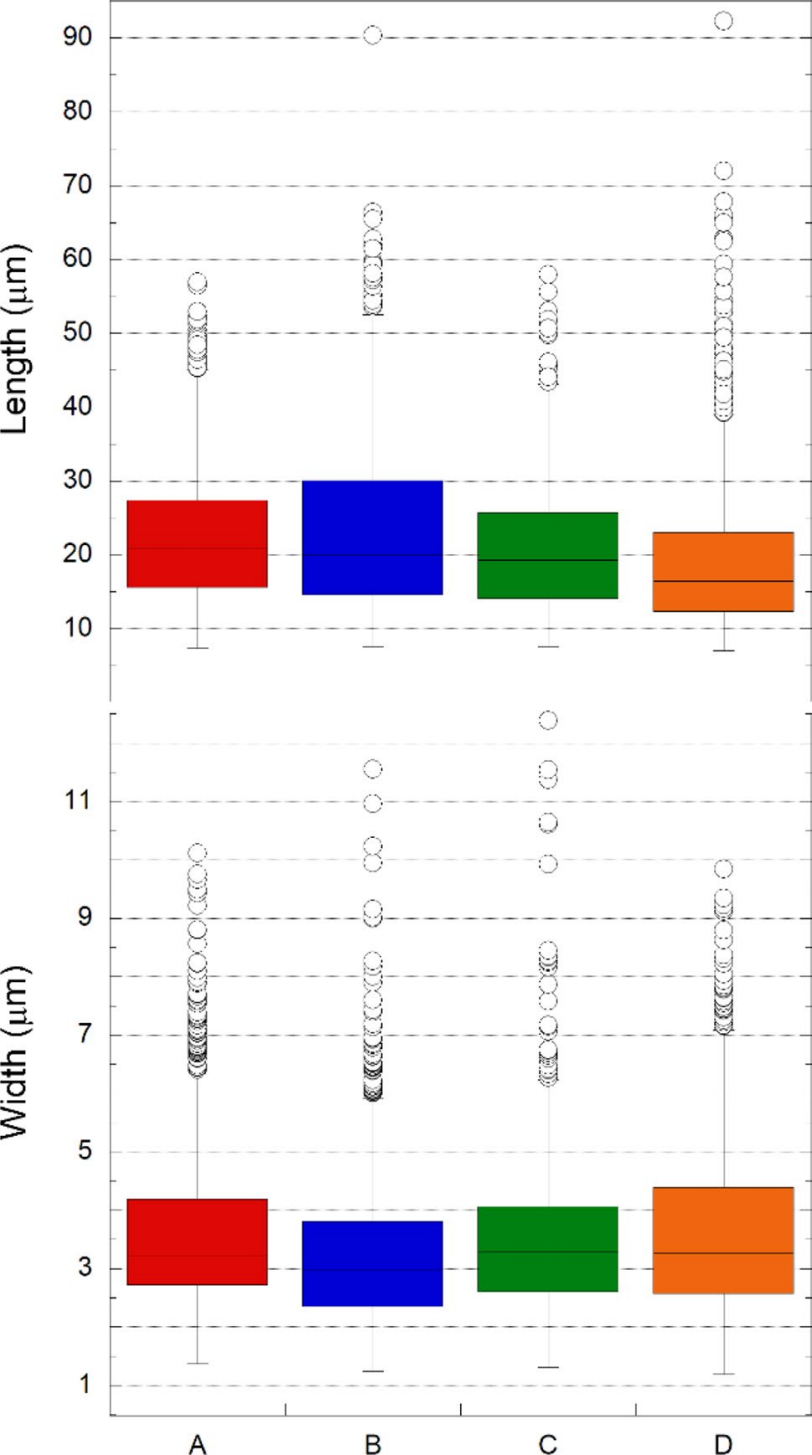
Box plots summarizing the distribution of the length (a) and width (b) values of the AB for samples A–C. Each box encloses 50% of the data with the median value of the variable displayed as a line. The top and bottom of the box mark the limits of ± 25% of the variable population (i.e. the Upper and Lower Quartiles, denoted as UpQ and LowQ, respectively). The lines extending from the top and bottom of each box mark the minimum and maximum values within the data set whose value is greater than UpQ + (1.5 × D_U-L_) or less than LowQ − (1.5 × D_U-L_), where D_U-L_ is the distance between UpQ and LowQ. Any value outside of this range (outlier) is displayed as an individual point.

The morphometry of each AB is automatically measured during the counting process with a precision corresponding to the size of the pixels (0.332 μm), as it is required by the counting routines to distinguish the AB from other particulate matter that may be present in the lungs (see “Materials and methods” section). This allows obtaining a complete knowledge on the distribution of the AB dimensions, i.e. average, median, and dispersion, which are summarized in Table 2 and, graphically, in Fig. 2. The results show that, regardless of the sample, the average length of the AB (longest axis) falls in a narrow range (19.4–23.6 μm). The length values maybe shifted towards higher values because, to filter out low aspect ratio (low length to width ratios) particulate matter, only objects with aspect ratio higher than 3 were considered. However, length values are well described by a log-normal distribution^41^, as expected assuming that they originate from the fragmentation of asbestos occurring during its extraction, industrial processing, and permanence in the lungs (Supplementary Fig. S1). The transversal dimensions of the AB (average of the two shorter axes) are distributed in an even narrower range (3.3–3.6 μm), reflecting the fact that the AB develop around mineral fibres of given diameter comprised in the range of about 0.1–0.4μm^42^.

## Discussion

Tomographic data provided tridimensional imaging of AB inside millimeter-size bulk lung tissue samples. The phase-contrast acquisition mode (opposed to conventional absorption acquisition) allowed revealing their spatial distribution and possible preferential accumulation in specific lung structures with unprecedented level of detail for 3D imaging. The tomograms revealed that the AB tend to accumulate at the end of alveolar sacs. In the same areas, hypercellularity is observed, which may be due to an accumulation of monocytes, as alveolar macrophages, in their attempt to engulf and remove the foreign fibres. The AB not only deposit on the walls of the sacs, but also tend to penetrate them, reaching the lung parenchyma.

The numbers of AB/g_dw_ calculated with the conventional approach (OM on tissue digests) were three order of magnitudes lower those calculated by XPCµT per unit volume (cm^3^) and then converted to g_dw_ using Eq. (1). This finding may indicate that conventional counting by OM severely underestimates the AB burden in lung tissues of exposed individuals. Indeed, AB quantification performed by SEM on samples A and B resulted in values that are closer to those obtained by XPCµT (Table 2). On the other hand, AB count by SEM performed on samples C and D resulted in even lower values than those estimated by OM.

Unfortunately, the lung tissue material was insufficient to perform triplicate AB count with conventional digestion and SEM and OM readings, as done for tomography counting. Therefore, no information is available on the variability of the AB numbers calculated with the conventional approach, which prevents finding a reliable relationship between the two counting methods. Indeed, one of the main problems of AB counting approaches is that they are based on the assumption that asbestos is uniformly distributed in the lungs. This, however, is not true, so that samples may not reflect the average asbestos burden in the lungs. For example, it is known that the lower lobes contain more asbestos, and variability of up to tenfold among different lung sections were observed^43^. However, even if the total volume probed with the conventional approach is larger than that probed by XPCµT (1 g of lung tissue roughly corresponds 1 cm^3^), only a small fraction of the area on which the AB are recovered (membrane or microscope slide) is read by SEM or OM to perform the counting. The result is then extended to the entire area, assuming uniform distribution. This may lead to a larger error with respect to the XPCµT approach, in which counting is performed over the entire volume imaged and without the approximations imposed by 2D readings. To obtain a reliable assessment of the AB burden in the lungs, the optimum would be to collect several lung tissue samples from different areas of the lower lobes. In this way, the average variability should not be more than two or threefold^21^. However, this approach is very time-consuming and only possible for autoptic (post-mortem) samples. On the other hand, XPCµT might enable the estimation of the AB burden also on living subjects through the collection of relatively non-invasive needle biopsy samples. Biopsies of non-neoplastic tissues for the lower lobes of either lungs might give a basic indication on the AB variability. From a practical point of view, the size and shape of needle biopsy samples (1–2 mm in diameter) is ideal for XPCµT imaging, as, for mathematical reasons concerning the reconstruction algorithms, the best shape of samples for X-ray tomography is a cylinder with a diameter slightly smaller than the X-ray beam spot size (field-of-view). Considering the most common experimental setup at synchrotron beamlines or laboratory X-ray sources (i.e. incident X-ray energies and detectors), samples of 1–2 mm in diameter are suitable for tomographic scanning. Considered also the average size and distribution of ABs, this sample size was found to be adequate to image a Representative Elementary Volume (REV)^44^ at sufficient spatial resolution (in general, in X-ray imaging the higher the resolution, the smaller is the area or volume that can be imaged^45^). To easily obtain almost perfect cylinders, a commercial tissue microarray (TMA)^46^ equipment, which is able to extract cores with few millimetre diameter from paraffin blocks, can be used. The acquisition of the data with a laboratory tomograph is then straightforward, as the samples can be mounted without further preparation and data acquisition takes few tens of minutes. Once the X-ray tomograms are acquired and reconstructed, the automated AB count takes few hours on a sufficiently fast computing infrastructure. For this proof-of-concept work the XPCµT data were acquired with the smaller voxel size available, which reflects in smaller probed volumes. However, test acquisitions (Supplementary Fig. S2) demonstrated that larger voxel sizes (0.8 × 0.8 × 0.8 µm^3^) and therefore larger probed volumes (2.05 × 2.05 × 1.73 mm^3^) could be used. This would ensure that commercial tomographs, which may have lower resolution, could indeed be used. Higher resolution reduces errors in the counting process. On the other side, larger volumes allow increasing the statistics and reducing the uncertainties arising from the heterogeneous distribution of the AB in the tissues. More experimental work is required to determine the optimal compromise between resolution and probed volume. Finally, the relatively long computational time required for the 3D counting process (hours) can be shortened by using dedicated software and optimizing the filter algorithms.

Asbestos bodies morphometry was naturally obtained during their count, and the average length of the AB was similar for all the studied samples. This information is useful to assess the health risk, as clearance mechanisms also depend on the fibres dimensions^22^. This may indicate that the asbestos fibres penetrating the lungs are subjected to the same fragmentation processes, regardless of the type of asbestos and its original length, at least when dealing with biopersistent amphiboles (which are believed to form the majority of the AB^47^). Accordingly, the distribution follows a log-normal curve (Supplementary Fig. S1), which is typical of fragmentation processes^41^. The observed morphometry of the AB is in agreement with the so called Stanton hypothesis, an empirical law stating that the AB preferentially develop on fibres longer than 10 μm and thinner than 0.5 μm^22^. An explanation of the Stanton hypothesis is that, due to the size of the human macrophages (~ 15–20 μm), only fibres longer than 10 μm can give rise to frustrated (uncomplete) phagocytosis that prevents their removal, eventually leading to chronic inflammation and triggering cytotoxic response.

As said above, the relatively low number of samples considered, prevented determining a reliable relationship between conventional and tomography counting methods, as the one determined by Roggli and Pratt^40^ between the number of AB in iron-stained histological sections and the AB concentration in digested lung tissue. For this reason, additional experimental work, considering a wider set of individuals with different degree of exposure to asbestos and a larger number of samples for each individual, is needed to fully validate the XPCµT method.

However, also considering these limitations, this novel approach is promising, as it possesses several advantages over the conventional approaches: (1) it can be applied to living subjects through relatively non-invasive lung biopsies; (2) the data acquisition is fast and straightforward and requires minimal sample preparation; (3) the counting process can be performed directly on the tomograms using automated routines, and is therefore less time consuming and reader/laboratory independent; (4) complete information on the AB morphometry is naturally derived during the counting process.

Besides the proposal of a novel, promising approach to assess asbestos burden in biological tissues, the present work also demonstrated the potential of advanced X-ray imaging (XPCµT) to reveal health-threatening fibres and particulate matter in biological tissues. The same techniques maybe used, for example, to study airborne particulate matter that is believed to be able to cross the brain barrier or the placenta, and that would be difficult to study by other means, without altering the samples.

## Materials and methods

Lung tissue samples were obtained *post-mortem* from the lower lobes of the lungs of four former workers with a history of occupational exposure to asbestos (steel plant, asbestos mine, and asbestos-cement product plant workers, see Table 1). Informed consent was obtained from the patients at the moment of their hospitalization and permission to perform research on human lung samples was obtained by the bioethical committees of the Martini hospital (Torino, Italy) and of the University of Torino (Turin, Italy). The authors declare that the research was carried out in accordance with the approved guidelines. The human lung samples used in this research are anonymous. Only non-neoplastic tissues were collected. All workers were subjected to prolonged exposure to asbestos (23.5 ± 10.5 years on average), and had severe-grade asbestos-related diseases (asbestosis, pleural-plaques, mesothelioma or lung cancer, Table 1). Subject D was also diagnosed with silicosis (exposure to silica powder) and siderosis (exposure to Fe-oxides powder), which are typical diseases of many industrial processes. The workers were exposed to an admixture of different asbestos phases, including amphiboles (mainly crocidolite and amosite) and serpentine (chrysotile), as asbestos often contains secondary asbestos phases (such as amphiboles contaminants in chrysotile batches).

The autoptic tissue samples were preserved in buffered formalin (10%) until measurements. The sample preparation for OM and SEM observations consisted of digesting respectively 1 g and 0.25 g of lung tissue with sodium hypochlorite (NaClO, 10%), and recovering the AB by filtration through microporous membranes (3 μm pore size for OM and 0.45 μm pore size for SEM). The AB count was carried out by observing, for OM and SEM respectively: (i) the whole membrane at × 400 magnification^48^; (ii) a portion of filter (representing 0.7% of the total area) at × 4000 magnification^34^ When sufficient material was available, a mass of 2.5 g of tissue was used to determine its dry weight, after dehydrating at 60 °C for 3 days. For all samples, the asbestos burden largely exceeded the amount established by the European Respiratory Society guidelines to indicate a high level of occupational exposure to asbestos (10^3^/g_dw_)^21^.

X-ray micro-tomography measurements were performed in phase-contrast mode (XPCµT) at the I13-2 beamline at the Diamond light source (UK), using a monochromatic incident X-ray energy of 14.7 keV. The phase-contrast mode allows imaging, at the same time, high and low absorbing materials (as the biological tissue and the AB) because the sensitivity to small density variations that can be measured by the phase variations of the X-rays can be orders of magnitude higher than conventional absorption variations^37^. Samples of suitable dimensions (1–2 mm in diameter, 10–15 mm height) were cut from the formalin-fixed paraffin-embedded tissue blocks using a scalpel, and mounted on the sample holder without further preparation. A PCOedge5.5 detector with pixel size 6.5 μm was used. The detector was coupled with a magnifying 20× optics, resulting in a sub-micron isotropic voxel size of 0.325 × 0.325 × 0.325 μm^3^. The detector array is composed by 2560 × 2560 pixels, thus a 3D volume of 2560 × 2560 × 2160 voxels was reconstructed, corresponding to a probed volume of 0.832 × 0.832 × 0.72 mm^3^. The volumes were stored in 32bit (real) images in TIF format. In-line free space propagation mode was used, i.e. the phase information was retrieved from the Fresnel’s diffraction patterns acquired at a defined sample to detector distance. To maximize the phase-contrast contribution, the samples were analysed at a distance of 50 mm from the detector. Several scans were performed in order to target different areas of the considered samples. For each tomogram, 2400 projections were acquired over a 0–180° rotation range, with 0.3 s dwell time (corresponding to a total acquisition time of ~ 12 min per tomography). A single-distance phase-retrieval algorithm proposed by Paganin et al.^49^ was applied. Data pre-processing, phase retrieval, and tomographic reconstruction were performed with the SYRMEP Tomo Project software^50^.

Automated software routines were used to estimate the number of AB per unit volume. To prevent underestimation errors due to the overlapping of the AB, counting was performed on volumes (3D) instead of their projection on a plane (2D).

**Figure 3:**
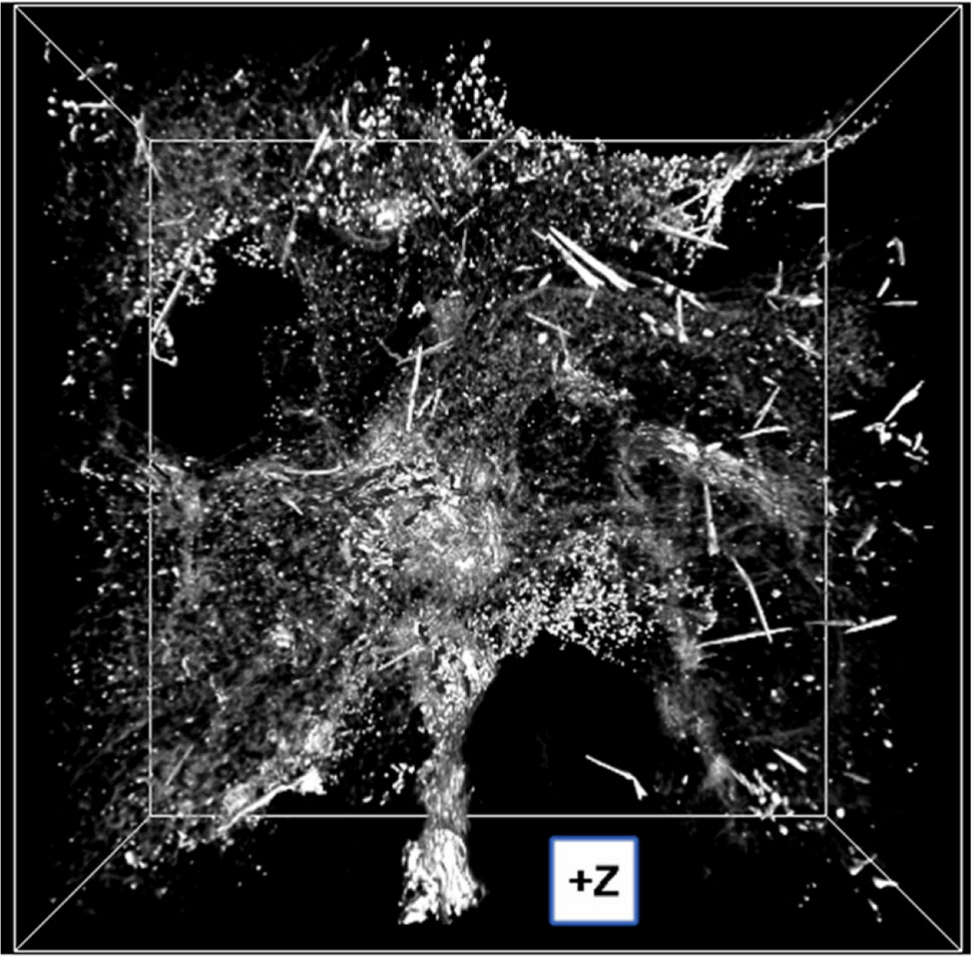
Perspective 3D view of a representative sub-volume of a lung tissue sample of size 0.495 × 0.495 × 0.132 mm^3^ (X, Y, Z) acquired at the I13-2 beamline at the Diamond Light Source (UK) at 14.7 keV incident X-ray energy. The bounding box is shown. The size of the (isotropic) voxels is 0.332 × 0.332 × 0.332 µm^3^.

For each sample, the average number of AB was estimated on sub-volumes of size 0.495 × 0.495 × 0.660 mm^3^, which, for computational reasons, were further divided in five sub-volumes of size 0.495 × 0.495 × 0.132 mm^3^ (Fig. 3). To introduce customized shape filters and have full control on the counting process, home-written MATLAB routines were used. The volumes were first segmented to separate the low X-ray absorbing background and biological tissue from higher absorbing objects (particulate matter and AB). Segmentation consists in selecting a range of pixel intensities (grey levels) including the objects of interest, as, physically, different pixels intensities correspond to different materials’ densities (in phase-contrast mode, denser materials result in higher (i.e. brighter) pixel grey levels). Then, shape filters based on the objects geometry (size, sphericity, and solidity) were applied. Minimum and maximum size filters (in voxels) were used to exclude, respectively, the smallest particulate matter, and some larger structures often present (probably pleural thickening), which have similar grey levels as the AB. Sphericity and solidity filters were used to detect objects with high aspect ratio among those who survived to the segmentation and size filter. The first is a measure of how much an objects resembles to a sphere (i.e. equal length of the x, y, and z axes). High aspect ratio objects, as AB, are expected to have low sphericity values. The solidity is defined as the ratio between the object volume and the volume of its convex hull (i.e. the volume underlying a surface wrapping the object). Quasi-cylindrical objects, as AB, are expected to have high solidity values.

Although the choice of the contrast and shape filters settings were critical for the correct estimation of the number of AB per unit volume, it was found that the same settings could be applied to all tomographic data acquired, regardless the Case examined. As the dimensions of the AB vary in a known range, the settings of the size and shape filters should not change considerably from sample to sample, though the presence and size of other absorbing particulate matter must be checked to avoid interference with the counting process. The choice of the contrast filter setting (grey level threshold), on the other hand, depends on the experimental setup used to acquire the tomographic data, and must therefore be carefully optimized for each instrument. The filter settings used in this work were the same for all datasets and are reported in Supplementary Table S1 as a reference.

**Figure 4:**
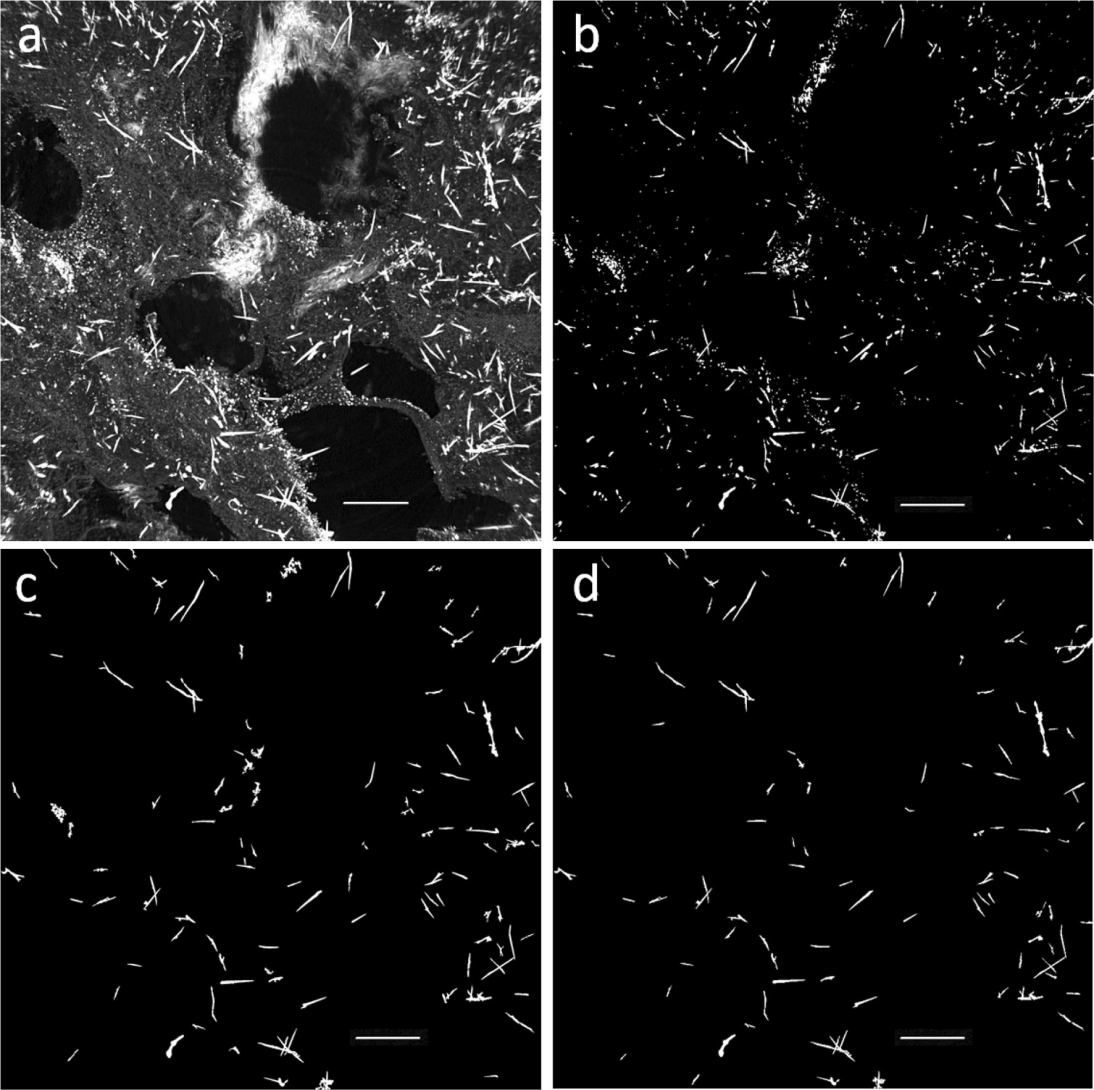
The counting process workflow is shown. (**a**) Original microtomography image projected on a plane for easy of view; (**b**) *Segmentation* most of the background lung tissue is removed only objects with grey levels in a chosen range survive; (**c**) *Size filters* only objects in a selected size range are selected, most of the smaller particulate matter or large/higher density background structures are removed; (**d**) *Sphericity and solidity filters* only objects with low sphericity and solidity values survive (i.e. objects with high aspect ratio are selected). The voxel size is 0.332 × 0.332 × 0.332 µm^3^ (isotropic), and the scale bars are 100 µm in length.

An example of the counting process workflow is shown in Fig. 4 on representative XPCµT data projected on a plane. Some AB in Fig. 4 appear to overlap with each other, but this is only because the imaged volume is projected on a plane. If 2D imaging were performed instead of tomography, the apparently overlapping AB would have been counted as one, and, for high asbestos burden samples, most of the AB would have appeared as overlapping. This well demonstrates the advantage of tomography (3D) over conventional imaging (2D). For each volume imaged, the tomographic reconstruction and counting process took several hours on a double-CPU (INTEL-XEON) machine with 256 Gb RAM.

## Supplementary Information

Supplementary Information.

## References

[1] Noonan CW (2017). Environmental asbestos exposure and risk of mesothelioma. Ann. Transl. Med..

[2] Uguen M (2017). Asbestos-related lung cancers: A retrospective clinical and pathological study. Mol. Clin. Oncol..

[3] Klebe S, Leigh J, Henderson DW, Nurminen M (2020). Asbestos, smoking and lung cancer: An update. Int. J. Environ. Res. Public Health.

[4] Jones RN, Hughes JM, Weill H (1996). Asbestos exposure, asbestosis, and asbestos-attributable lung cancer. Thorax.

[5] Mazurek JM, Syamlal G, Wood JM, Hendricks SA, Weston A (2017). Malignant mesothelioma mortality—United States, 1999–2015. Morb. Mortal. Wkly. Rep..

[6] Kameda T (2014). Asbestos: Use, bans and disease burden in Europe. Bull. World Health Organ..

[7] Park EK (2011). Global magnitude of reported and unreported mesothelioma. Environ. Health Perspect..

[8] Carbone M (2019). Mesothelioma: Scientific clues for prevention, diagnosis, and therapy. CA Cancer J. Clin..

[9] Bianchi C, Bianchi T (2014). Global mesothelioma epidemic: Trend and features. Indian J. Occup. Environ. Med..

[10] Abdel-Rahman O (2018). Global trends in mortality from malignant mesothelioma: Analysis of WHO mortality database (1994–2013). Clin. Respir. J..

[11] Carbone M (2012). Malignant mesothelioma: Facts, myths, and hypotheses. J. Cell. Physiol..

[12] Reid A (2014). Mesothelioma risk after 40 years since first exposure to asbestos: A pooled analysis. Thorax.

[13] Mace ML, McLemore TL, Roggli V, Brinkley BR, Greenberg SD (1980). Scanning electron microscopic examination of human asbestos bodies. Cancer Lett..

[14] McLemore TL (1980). Asbestos body phagocytosis by human free alveolar macrophages. Cancer Lett..

[15] Governa M (1999). Role of iron in asbestos-body-induced oxidant radical generation. J. Toxicol. Environ. Heal. A.

[16] Ghio AJ, Stonehuerner J, Richards J, Devlin RB (2008). Iron homeostasis in the lung following asbestos exposure. Antioxid. Redox Signal..

[17] Lund LG, Williams MG, Dodson RF, Aust AE (1994). Iron associated with asbestos bodies is responsible for the formation of single strand breaks in phi X174 RFI DNA. Occup. Environ. Med..

[18] Hardy JA, Aust AE (1995). The effect of iron binding on the ability of crocidolite asbestos to catalyze DNA single-strand breaks. Carcinogenesis.

[19] Fubini B, Mollo L (1995). Role of iron in the reactivity of mineral fibers. Toxicol. Lett..

[20] Churg AM, Warnock ML (1981). Asbestos and other ferruginous bodies: Their formation and clinical significance. Am. J. Pathol..

[21] De Vuyst P (1998). Guidelines for mineral fibre analyses in biological samples: Report of the ERS Working Group. European Respiratory Society. Eur. Respir. J..

[22] Stanton MF (1981). Relation of particle dimension to carcinogenicity in amphibole asbestoses and other fibrous minerals. J. Natl. Cancer Inst..

[23] Feder IS (2017). The asbestos fibre burden in human lungs: new insights into the chrysotile debate. Eur. Respir. J..

[24] Visonà SD (2021). Inorganic fiber lung burden in subjects with occupational and/or anthropogenic environmental asbestos exposure in broni (Pavia, northern italy): An sem-eds study on autoptic samples. Int. J. Environ. Res. Public Health.

[25] Churg A (1994). Deposition and clearance of chrysotile asbestos. Ann. Occup. Hyg..

[26] Dumortier P, De Vuyst P, Strauss P, Yernault JC (1990). Asbestos bodies in bronchoalveolar lavage fluids of brake lining and asbestos cement workers. Br. J. Ind. Med..

[27] Cruz MJ (2017). Utility of bronchoalveolar lavage for the diagnosis of asbestos-related diseases. Arch. Bronconeumol..

[28] Karjalainen A, Nurminen M, Vanhala E, Vainio H, Anttila S (1996). Pulmonary asbestos bodies and asbestos fibers as indicators of exposure. Scand. J. Work. Environ. Health.

[29] Nuyts V, Vanhooren H, Begyn S, Nackaerts K, Nemery B (2017). Asbestos bodies in bronchoalveolar lavage in the 21st century: A time-trend analysis in a clinical population. Occup. Environ. Med..

[30] Velasco-García MI (2011). Reproducibility of asbestos body counts in digestions of autopsy and surgical lung tissue. Am. J. Ind. Med..

[31] Teschler H, Thompson AB, Dollenkamp R, Konietzko N, Costabel U (1996). Relevance of asbestos bodies in sputum. Eur. Respir. J..

[32] Bardelli F (2017). New insights on the biomineralisation process developing in human lungs around inhaled asbestos fibres. Sci. Rep..

[33] Borelli V (2007). A procedure for the isolation of asbestos bodies from lung tissue by exploiting their magnetic properties: A new approach to asbestos body study. J. Toxicol. Environ. Health A.

[34] Belluso E (2006). Assessment of inorganic fibre burden in biological samples by scanning electron microscopy—Energy dispersive spectroscopy. Microchim. Acta.

[35] Di Giuseppe D (2019). Mineral fibres and asbestos bodies in human lung tissue: A case study. Minerals.

[36] Craighead JE (1982). The pathology of asbestos-associated diseases of the lungs and pleural cavities: Diagnostic criteria and proposed grading schema. Report of the Pneumoconiosis Committee of the College of American Pathologists and the National Institute for Occupational Safety and Health. Arch. Pathol. Lab. Med..

[37] Khimchenko A (2018). Hard X-ray nanoholotomography: Large-scale, label-free, 3D neuroimaging beyond optical limit. Adv. Sci..

[38] Pooley FD, Ranson DL (1986). Comparison of the results of asbestos fibre dust counts in lung tissue obtained by analytical electron microscopy and light microscopy. J. Clin. Pathol..

[39] Davis JM (1964). The ultrastructure of asbestos bodies from human lung. Br. J. Exp. Pathol..

[40] Roggli VL, Pratt PC (1983). Numbers of asbestos bodies on iron-stained tissue sections in relation to asbestos body counts in lung tissue digests. Hum. Pathol..

[41] Newman MEJ (2005). Power laws, Pareto distributions and Zipf’s law. Contemp. Phys..

[42] Cluff DL, Patitsas AJ (1992). Size characterization of asbestos fibers by means of electrostatic alignment and light-scattering techniques. Aerosol Sci. Technol..

[43] Gibbs AR, Pooley FD (1996). Analysis and interpretation of inorganic mineral particles in ‘lung’ tissues. Thorax.

[44] Hill R (1963). Elastic properties of reinforced solids: Some theoretical principles. J. Mech. Phys. Solids.

[45] Buzug T (2008). Computed Tomography: From Photon Statistics to Modern Cone-Beam CT.

[46] Jawhar NMT (2009). Tissue microarray: A rapidly evolving diagnostic and research tool. Ann. Saudi Med..

[47] Giacobbe C (2021). Crystal structure determination of a lifelong biopersistent asbestos fibre using single-crystal synchrotron X-ray micro-diffraction. IUCrJ.

[48] Karjalainen A (1996). Asbestos bodies in bronchoalveolar lavage in relation to asbestos bodies and asbestos fibres in lung parenchyma. Eur. Respir. J..

[49] Paganin D, Mayo SC, Gureyev TE, Miller PR, Wilkins SW (2002). Simultaneous phase and amplitude extraction from a single defocused image of a homogeneous object. J. Microsc..

[50] Brun F (2017). SYRMEP Tomo project: A graphical user interface for customizing CT reconstruction workflows. Adv. Struct. Chem. Imaging.

