# Supplementary material for: Asbestos bodies count and morphometry in bulk lung tissue samples by non-invasive X-ray micro-tomography
Source: Sci Rep. 2021 May 19;11:10608. doi: 10.1038/s41598-021-90057-1 (PMC8136473; doi:10.1038/s41598-021-90057-1)
Supplement: Supplementary file 1 — Supplementary Information. [file 41598_2021_90057_MOESM1_ESM.docx]

**Asbestos bodies count and morphometry in bulk lung tissue samples by non-invasive x-ray micro-tomography**

Supplementary Information


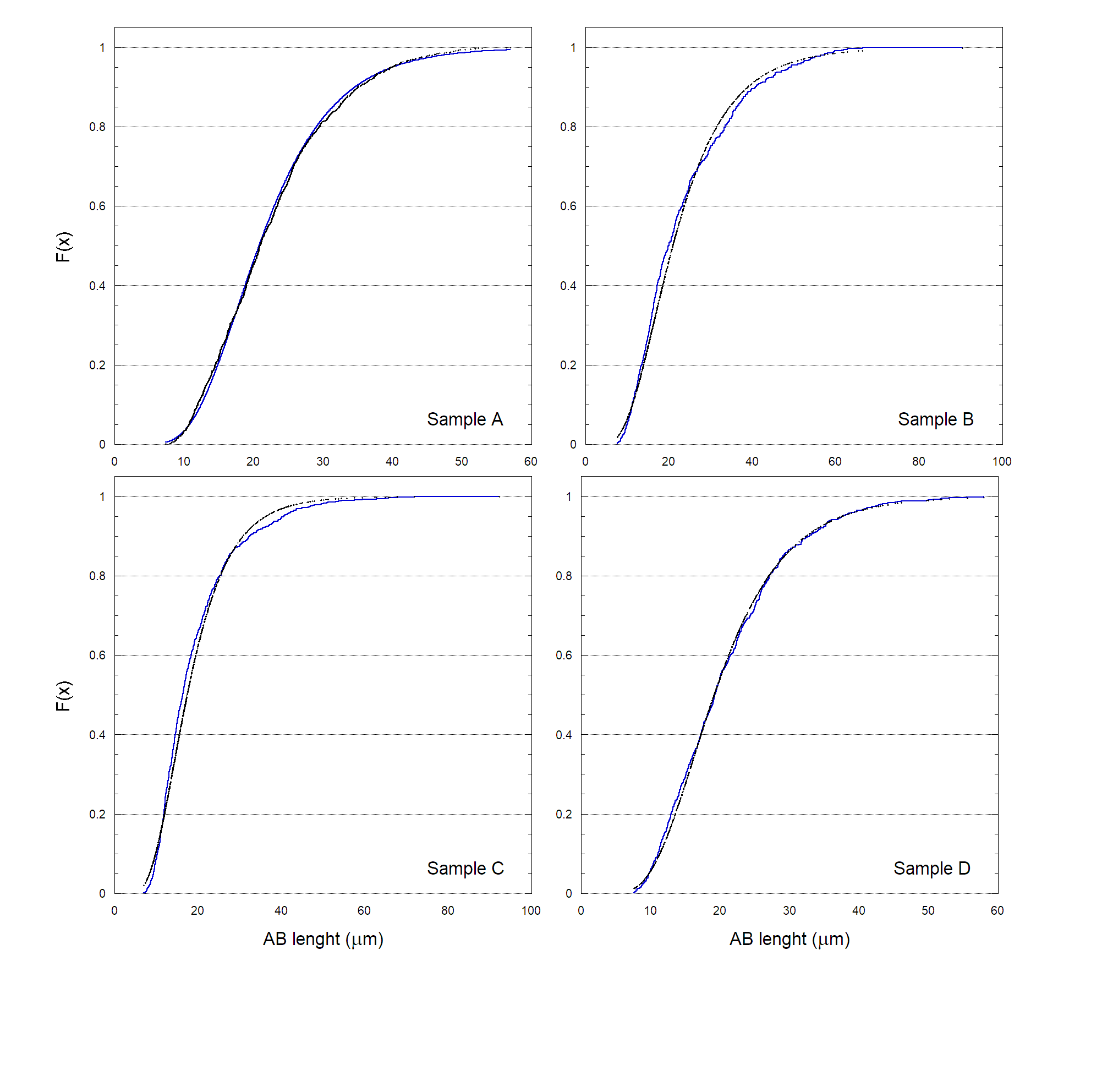


**Supplementary Figure 1.** The panels show the cumulative distribution functions of the AB length values of all samples (black points). The experimental points are in excellent agreement with the log-normal distribution cumulative functions (blue solid lines), which are typical of fragmentation processes (see main text) and are described by the following equation:

$$F\left( x \right)=\frac{1}{2}+\frac{1}{2}\frac{\mathrm{erf} \left( \ln\left( x \right)- \mu\right)}{\surd2}$$

where µ and σ are the experimental means and standard deviations of the logarithm of the length values.

**

**

**Supplementary Figure 2.** Test XPCµT image of a lung tissue portion projected on a plane for easy of view. The tomographic data were acquired at the I13-2 beamline at the Diamond light source (UK) at 14.7 keV incident x-ray energy, using a voxel sizes is 0.8 x 0.8 x 0.8µm^3^. The probed volume shown is 2.05 x 2.05 x 1.73 mm^3^ in size. Asbestos bodies are clearly visible as the white, high aspect ratio objects.

**Supplementary Table 1.** Final size, shape, and contrast filter settings used for all datasets. The size filter setting are in voxels (one voxel corresponds to 0.332 x 0.332 x 0.332 µm^3^), and the shape and contrast filter settings are dimensionless. Minimum size: objects with voxel size smaller than the setting were not included in the count. Maximum size: objects with voxel size larger than the setting were not included in the count. Sphericity: objects with a sphericity value higher than the setting (i.e. more spherical) were not included in the count. The Solidity filter was not used in final counts as it was found to filter out very few objects, and slowed the counting process. Contrast filter: objects with grey levels lower than the setting were not included in the count. *In phase-contrast mode, denser materials correspond to higher grey levels. The threshold value refers to real (float) 32bit TIF images, and it is specific to the experimental setup used.

| Size filters | | Shape filters | | Contrast filter^*^ |
| --- | --- | --- | --- | --- |
| Minimum size | Maximum size | Sphericity | Solidity | Grey level threshold |
| 500 | 10,000 | 0.65 | Not used | 0.0035 |
